# Type of sweet flavour carrier affects thyroid axis activity in male rats

**DOI:** 10.1007/s00394-016-1367-x

**Published:** 2016-12-31

**Authors:** Ewelina Pałkowska-Goździk, Anna Bigos, Danuta Rosołowska-Huszcz

**Affiliations:** Department of Dietetics, Faculty of Human Nutrition and Consumer Sciences, University of Life Sciences in Warsaw-SGGW, Poland, Nowoursynowska 159c Street, 02-776 Warsaw, Poland

**Keywords:** Thyroid hormones, Sucrose, Non-nutritive sweeteners, Sucralose

## Abstract

**Purpose:**

Non-nutritive sweeteners are the most widely used food additives worldwide. However, their metabolic outcomes are still a matter of controversy and their effect on the thyroid activity, a key regulator of metabolism, has not been previously studied. Therefore, we aim to determine the influence of the sweet type flavour carrier on selected parameters of thyroid axis activity.

**Methods:**

Male Sprague–Dawley rats (*n* = 105) were divided into 3 groups fed ad libitum for three weeks isocaloric diets (3.76 ± 0.5 kcal/g): two with the same sweet flavour intensity responded to 10% of sucrose (with sucrose–SC—and sucralose—SU) and one non-sweet diet (NS). To evaluate the post-ingested effects, animals were euthanised at fast and 30, 60, 120, 180 min after meal.

**Results:**

The results obtained indicate that both the presence and the type of sweet taste flavour carrier affect thyroid axis activity both at fasting and postprandial state. Compared to diet with sucrose which stimulates thyroid axis activity, sucralose addition diminishes thyroid hormone synthesis as thyroid peroxidase (TPO) activity, plasma thyroxine (T4), and triiodothyronine (T3) concentration was lower than in SC and NS while in non-sweet diet the lowest level of hepatic deiodinase type 1 (DIO1) and the highest reverse T3 (rT3) level indicate on altered thyroid hormone peripheral metabolism.

**Conclusion:**

Both the presence and the type of sweet flavour carrier have a significant impact on thyroid axis activity. Our findings suggest that this organochlorine sweetener is metabolically active and might exacerbate metabolic disorders via an adverse effect on thyroid hormone metabolism.

## Introduction

The adaptive evolutionary development resulted in a distinct preference for sweet-tasting foods as a desirable source of energy [1]. Facing the fact that an innate desire for sweetness has been linked to the global rising rates of obesity and comorbidities, non-nutritive sweeteners (NNS) have been proposed as a useful tool for both prevention and dietary interventions in the treatment of those diet-related disorders [2].

However, the impact of artificial sweeteners on human health raises a number of concerns [3, 4]. There is accumulating evidence that the discrepancy between a sweet taste and the expected energy dose could have hormonal, metabolic, and thermogenic consequences [5]. Adverse effects of NNS consumption on the energy balance leading to hyperphagia and weight gain have been reported [6–9] although the mechanisms of their occurrence are not yet fully characterised [2, 9].

Despite the presumed effect of artificial sweeteners on the body energy balance, their impact on thyroid activity has not been investigated. Thyroid hormones (TH) play an essential role in maintaining energy homoeostasis through both central and peripheral pathways [10]. They are involved in the regulation of protein, fat, and carbohydrate metabolism; thus, both the quantity and quality of the diet affect hypothalamic-pituitary-thyroid (HPT) axis activity [11, 12]. Circulating levels of thyroid hormones are impacted by the consumption of carbohydrates [13, 14]. According to Shafrir [13], a sucrose-rich diet accelerated T4 to T3 conversion and induced the elevation of T3, while the deprivation of carbohydrates decreased plasma T3 concentration [14]. Early studies on the relationship between nutrition and thyroid function provided evidence that only diets containing carbohydrates were able to normalise the plasma thyroid hormone profile after long-term fasting [15].

Therefore, we aimed to compare the effects of isocaloric sweetness-matched diets with sucrose and sucralose on thyroid activity. Sucralose is a chlorinated disaccharide with the chemical formula 1,6-dichloro-1,6-dideoxy-β-D-fructofuranosyl-4-chloro-4-deoxy-α-D-galactopyranoside. Sucralose is authorised in the European Union for food use; its safety has been extensively evaluated, and an acceptable daily intake (ADI) of 15 mg/kg of body weight was established by the EU Scientific Committee on Food. Sucralose is approximately 600 times sweeter by weight than sucrose and, among commonly used NNS, the flavour profile of sucralose is the most similar to that of sucrose [16]. Due to its physicochemical properties, sucralose is found in thousands of beverages, food, and pharmaceutical products as a sugar substitute [17].

In vitro studies have demonstrated that sucralose and sucrose stimulate the same sweet taste G protein-coupled receptor complex T1R2/T1R3 in the oral cavity of humans, pigs, rats, and mice [18]. Studies of absorption, distribution, and metabolism of sucralose suggest that the pharmacokinetics of sucralose is similar in humans and rats, although the threshold of sweetness acceptance of sucralose is different [19]. Over the past few years, the body of data on taste-related signalling molecules found in extraoral tissue has grown constantly [20, 21]. Sweet taste receptors are expressed in the gastrointestinal tract, pancreas, brain, and adipose tissue where they are presumed to be involved in nutrient-sensing and energy metabolism regulation [22]. Moreover, T1R2 and T1R3 are expressed in tissues wherein their function appears less evident, including those of bladder, heart, and testis [20, 21]. Sweet tastants might also exert an important regulatory role in thyroid axis activity since G protein-coupled bitter taste receptors were also found in the thyroid gland where they appear to mediate a protective response to endocrine disruptors by regulating thyrocyte function and hormone production [23].

The purpose of this study was to determine the effect of a sweet-tasting diet on selected parameters of thyroid axis activity including the concentration of the plasma thyroid-stimulating hormone (TSH), thyroxine, triiodothyronine, both total and free (T4, T3 and fT4, fT3, respectively), reverse triiodothyronine (rT3), as well as thyroid peroxidase (TPO) and hepatic deiodinase type 1 (DIO1) content, using a rodent model. Three types of diets were tested: (1) a diet sweetened with a caloric sweetener—sucrose, (2) a diet sweetened with an artificial organochlorine sweetener—sucralose, and (3) a non-sweet diet control.

## Materials and methods

### Animals and diets

The experiment was carried out on 105 8-week-old male Sprague–Dawley rats with an average initial body weight of 325 ± 19 g obtained from the Medical Research Centre of the Polish Academy of Sciences (Warsaw, Poland).

Animals were individually housed under controlled environmental conditions (12-h light: 12-h darkness cycle, temperature 22 ± 1 °C, and 60% humidity) with ad libitum access to standard rodent chow (Labofeed H) and fresh water. After a 7-day adaptation period, animals (*n* = 105) were randomly assigned to three groups and for three weeks fed ad libitum semisynthetic (based on AIN-93-M diet formula) isocaloric diets (3.76 ± 0.5 kcal/g): two with the same sweetness intensity equivalent to 10 g of sucrose per 100 g of food (with sucrose—SC—and sucralose—SU) and one non-sweet diet (NS). Diet composition is presented in Table 1.

**Table 1:** Composition of experimental diets (g/100 g)

Ingredients	NS	SC	SU
Wheat starch	63.5	53.5	63.48
Sucrose	0	10	0
Sucralose	0	0	0.0167
Casein	20.0	20.0	20.0
Soybean oil	7.0	7.0	7.0
Potato starch	5.0	5.0	5.0
Mineral mixture*	3.5	3.5	3.5
Vitamin mixture	1.0	1.0	1.0

*NS* non-sweet diet, *SC* diet with sucrose, *SU* diet with sucralose

* Mineral and vitamin mixtures were provided by Sigma-Aldrich (USA)

Food intake was recorded daily and body weight three times per week. At the end of the experiment, animals were euthanised after 16-hour fast and 30, 60, 120, 180 min after a meal corresponding to the examined group (7 rats from each group/time point). Blood was collected by cardiac puncture. Thyroids and livers were taken out and immediately frozen in liquid nitrogen. Blood serum, thyroids, and livers were stored at −80 °C until further analysis. The study protocol was approved by the 3rd Local Ethical Commission in Warsaw (no. 27/2009), and the study was conducted in compliance with applicable national laws and regulations.

### Biochemical analysis

The concentrations of the plasma thyroid-stimulating hormone (TSH), thyroxine, triiodothyronine, both total and free (T4, T3, and fT4, fT3, respectively), and reverse triiodothyronine (rT3) were measured by radioimmunoassay using commercial kits (IZOTOP, Institute of Isotopes Ltd., Budapest, Hungary). All procedures were carried out according to provided kit instructions. Hormone determinations were made in triplicate. The sensitivity for TSH, T4, T3, fT4, fT3, and rT3 was 0.5 ng/ml, 7 nmol/l, 0.3 nmol/l, 0.7 nmol/l, 0.58 pmol/l, 0.009 ng/ml, respectively.

Thyroid peroxidase (TPO) preparation and analysis were done using the method described by Hosoya et al. [24]. Enzyme activity from the thyroid microsomal fraction was determined spectrophotometrically at 350 nm by measuring the rate of oxidation of iodide to iodine in the presence of hydrogen peroxide as a second substrate. The measurement was performed at room temperature (21 ± 1 °C), and the absorbance was measured every 8 s for 160 s. The reaction mixture contained the enzyme preparation (40–80 μg), phosphate buffer (50 mM, pH = 7.4), hydrogen peroxide (135 mM), and potassium iodide (13 mM). Protein concentration was measured by the method of Bradford (Merck, Germany) with bovine serum albumin (BSA) as a standard (Sigma-Aldrich, USA). A TPO activity unit was defined as the change in the absorbance of 0.001 per second per 1 mg of protein in the enzyme preparation (mU/s/mg protein).

The level of deiodinase type 1 (DIO1) protein in liver homogenates was measured by the sandwich immunoassay method validated for use in rats (USCN Life Science Inc., China) according to the manufacturer’s instructions. The DIO1 level was expressed as ng per mg liver homogenate protein determined by the Bradford method with BSA as a standard (Sigma-Aldrich, USA). The minimum detectable dose of rat DIO1 was less than 0.065 ng/ml. No significant cross-reactivity or interference was observed with deiodinase type 1.

### Statistical analysis

Results are presented as mean ± SEM. Two-way ANOVA was used to analyse changes in food ingestion, body weight, and the effects of the diet and time, followed by the post hoc LSD Fisher’s test. Differences were considered to be significant at *p* < 0.05. All statistical analyses were performed using the Statistica v10 software package (StatSoft, USA).

## Results

### Food intake and body weight gain

Both food intake and body mass gain were significantly affected by the type of diet (for both *p* < 0.001). In total, the highest food intake was recorded in the SU group. The average daily intake of sucralose with the diet (14.2 ± 0.4 mg/kg body weight/day) did not exceed the acceptable daily intake (ADI, 15 mg/kg body weight/day).

The food intake recorded during the meal before euthanasia did not differ between NS, SC, and SU, and was 3.98 ± 0.5, 4.22 ± 0.41, and 4.71 ± 0.5, respectively.

The total daily body weight gain in the SU group was significantly higher than in SC and NS, which represented the lowest value (for both *p* < 0.001). Therefore, the highest diet growth efficiency was also recorded in SU, and there were no differences between NS and SC (Table 2).

**Table 2:** Food intake, body weight, and thyroid mass to body weight index across dietary groups

Monitored parameters	Dietary groups		
	NS	SC	SU
Food intake (g/day)	25.01 ± 0.26^A^	29.15 ± 0.16^B^	31.18 ± 0.26^C^
Body weight gain (g/day)	1.58 ± 0.06^A^	2.00 ± 0.05^B^	2.58 ± 0.05^C^
Diet growth efficiency (g/day)*	0.06 ± 0.003^A^	0.07 ± 0.002^A^	0.09 ± 0.002^B^
Thyroid/body weight (mg/100 g body weight)	5.22 ± 0.43	5.79 ± 0.36	6.21 ± 0.33

Data presented as mean ± SEM

*NS* non-sweet diet, *SC* diet with sucrose, *SU* diet with sucralose

* Diet growth efficiency—body weight gain (g/day) to food intake (g/day) ratio

A, B, C letters indicate significant differences between dietary groups (*p* < 0.05)

The thyroid to body weight index did not differ among the groups (Table 2).

### TSH, TPO activity, and plasma thyroid hormone concentrations

**Fig. 1:**
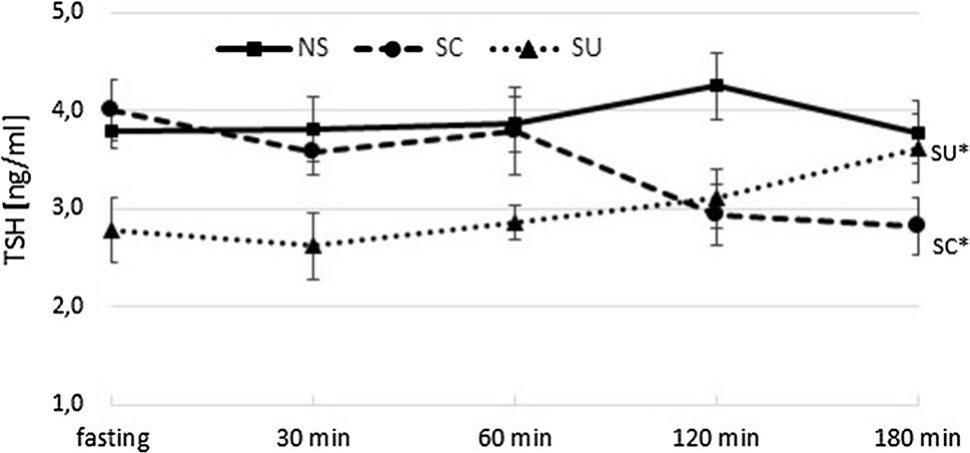
Plasma TSH concentration (ng/ml) at fasting, 30, 60, 120, 180 min after the meal; *NS* non-sweet diet, *SC* diet with sucrose, *SU* diet with sucralose; data presented as mean ± SEM; *p < 0.05 compared to baseline

The plasma TSH level was significantly influenced by the diet type (*p* < 0.001) as shown in Fig. 1. The interactions between the diet type and time after meal ingestion were also shown (*p* = 0.002). At fasting, TSH concentration in the SU group was significantly lower than in the two other groups (for both *p* < 0.001). Food intake did not affect TSH concentrations in NS, while postprandial TSH changes in SC were different from those observed in SU. After 180 min, a 30% decrease in SC and the same degree of TSH increase in SU were noted (180 min vs baseline, *p* < 0.001 and *p* = 0.014, respectively) (Fig. 1).

**Fig. 2:**
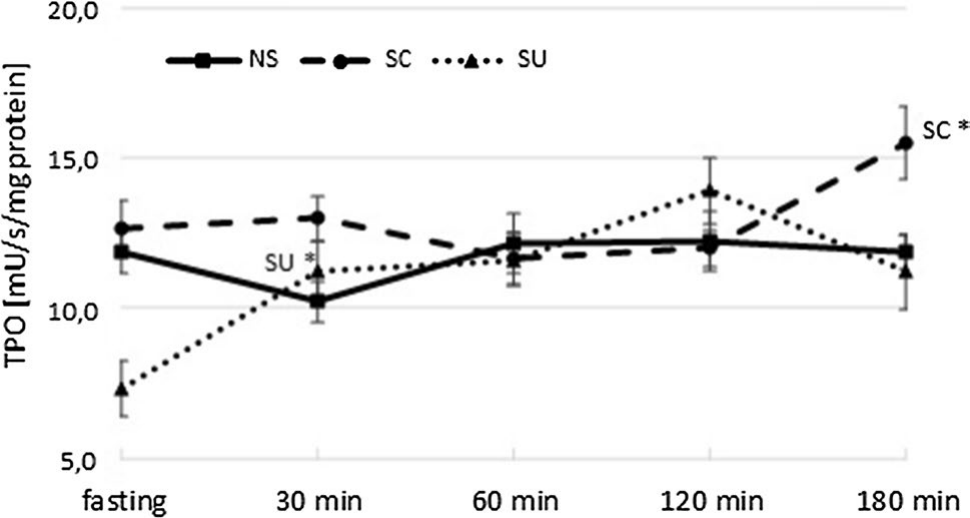
TPO activity (mU/s/mg protein) at fasting and 30, 60, 120, 180 min after the meal; NS, non-sweet diet; SC, diet with sucrose; SU, diet with sucralose; data presented as mean ± SEM; *p < 0.05 compared to baseline

TPO activity was significantly influenced by the type of diet (*p* < 0.001) and time after the meal (*p* < 0.001). At fasting, TPO activity was significantly lower in SU than in the SC and NS groups (for both *p* < 0.001). Food ingestion caused an increase in TPO activity only in the sweet-tasting diets observed at different time points—after 30 min in SU (30 min vs baseline, *p* = 0.004) and after 180 min in SC (180 min vs baseline, *p* = 0.033). In SC, TPO activity at the end of the sampling period remained higher than the values in SU and NS (for both *p* < 0.001) (Fig. 2).

**Fig. 3:**
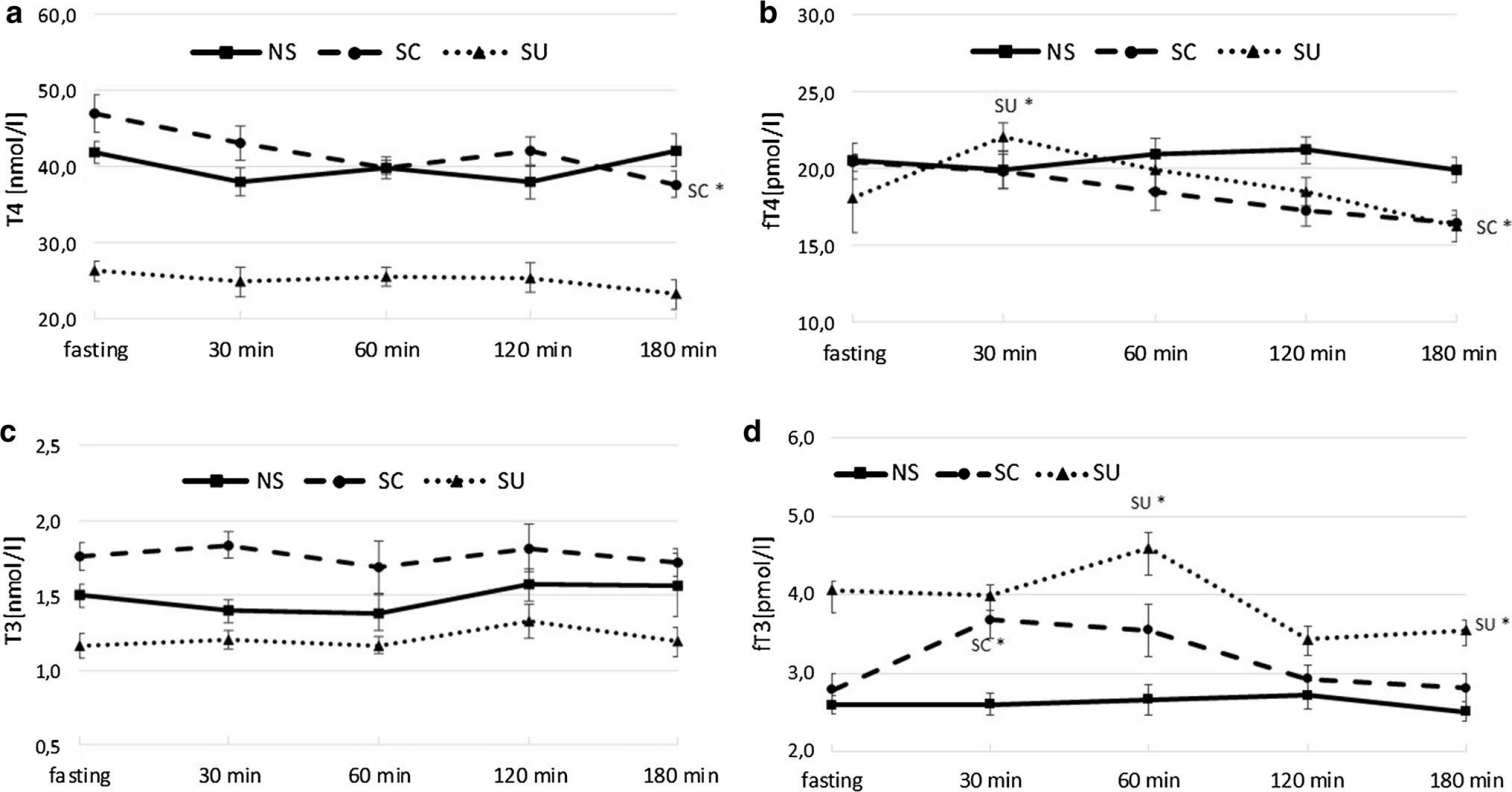
Plasma thyroid hormone concentration at fasting and 30, 60, 120, 180 min after the meal; NS, non-sweet diet; SC, diet with sucrose; SU, diet with sucralose; **a** thyroxine concentration (nmol/l); **b** free thyroxine concentration (pmol/l); **c** triiodothyronine concentration (nmol/l); **d** free triiodothyronine concentration (pmol/l); data presented as mean ± SEM; *p < 0.05 compared to baseline

For T4 concentration, the effects of the diet type (*p* < 0.001) and time after the meal (*p* < 0.001) as well as the interaction between both factors (*p* < 0.001) were significant. The triiodothyronine level was affected solely by the diet type (*p* < 0.001). At fast, T4 and T3 in SC were higher than in NS (*p* = 0.033 and *p* = 0.021, respectively) and SU (for both *p* < 0.001). Postprandial T4 changes were observed only in the SC group where the T4 level decreased significantly 180 min after the meal (180 min vs baseline *p* = 0.015). During the entire sampling period, T4 levels in SU were lower than in NS and SC (for both *p* < 0.001). There were no significant postprandial alterations in T3 concentration in the experimental groups, and in SU the hormone level remained significantly lower than in NS and SC at each time point (for all *p* < 0.05) (Fig. 3a, c).

Baseline fT4 levels did not differ among the groups. Food intake affected the fT4 level in the groups receiving the sweetened diets, but the pattern differed between those groups. In SU, a significant increase occurred 30 min after the meal (30 min vs baseline *p* = 0.031), while in SC, an evident decrease was observed after 180 min (180 min vs baseline *p* = 0.025). At the end point, fT4 levels in SC and SU were lower than in NS (*p* = 0.043 and *p* = 0.035, respectively) (Fig. 3b).

The fasting fT3 plasma level in SU was higher than in the other groups (for both *p* < 0.001). The feeding caused a significant increase in fT3 concentration in the groups fed sweetened diets. In SC, the increase occurred after 30 min (*p* = 0.002), and in SU it was observed 30 min later (*p* = 0.027). At the end of the sampling period, the fT3 level in SU remained higher than in the two other groups (for SC *p* = 0.040, for NS *p* = 0.018) (Fig. 3d). There were no significant post meal changes in fT3 concentration in rats fed the non-sweet diet; however, after 180 min, the hormone level was not different from the SC value

**Fig. 4:**
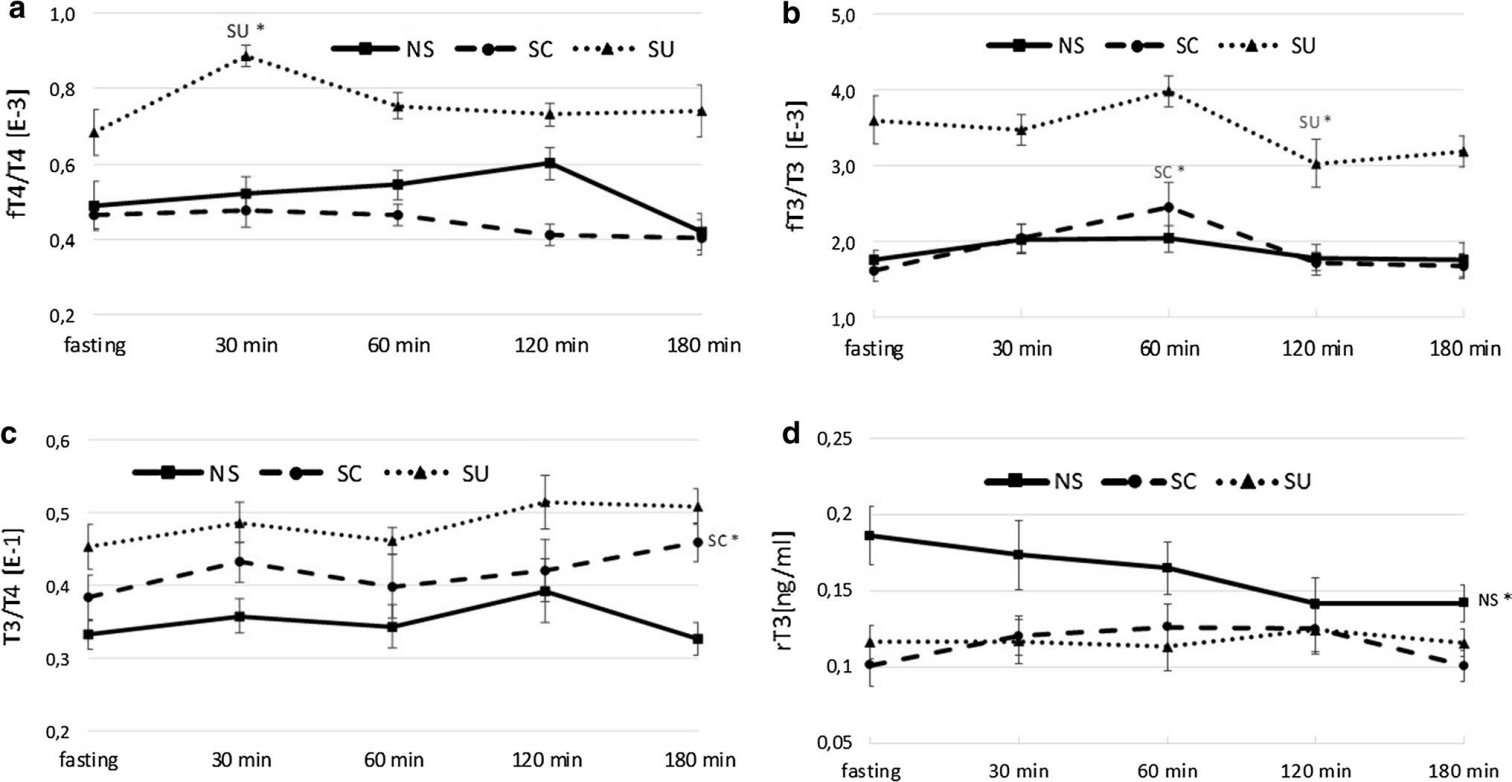
**a** fT4/T4 index; **b** fT3/T3 index; **c** T3/T4 index; **d** rT3 (ng/ml) at fasting and 30, 60, 120, 180 min after the meal; NS, non-sweet diet; SC, diet with sucrose; SU, diet with sucralose; data presented as mean ± SEM; *p < 0.05 compared to baseline

Both at fasting and postprandially, fT4/T4 and fT3/T3 in SU were significantly higher than in NS and SC (*p* < 0.05 for all) (Fig. 4a, b). At fast and at the end point, the T3/T4 ratio remained significantly higher in SU than in NS (*p* = 0.017), but not different from the ratio noted in SC (Fig. 4c).

At fasting, rT3 concentration in NS was higher than in SC and SU (for both *p* < 0.001) and despite a feeding-induced decline remained the highest after 180 min (*p* = 0.038 and *p* = 0.045, respectively). There were no postprandial changes in rT3 in the groups fed the sweetened diets (Fig. 4d).

### Deiodinase type 1 level in the liver

**Fig. 5:**
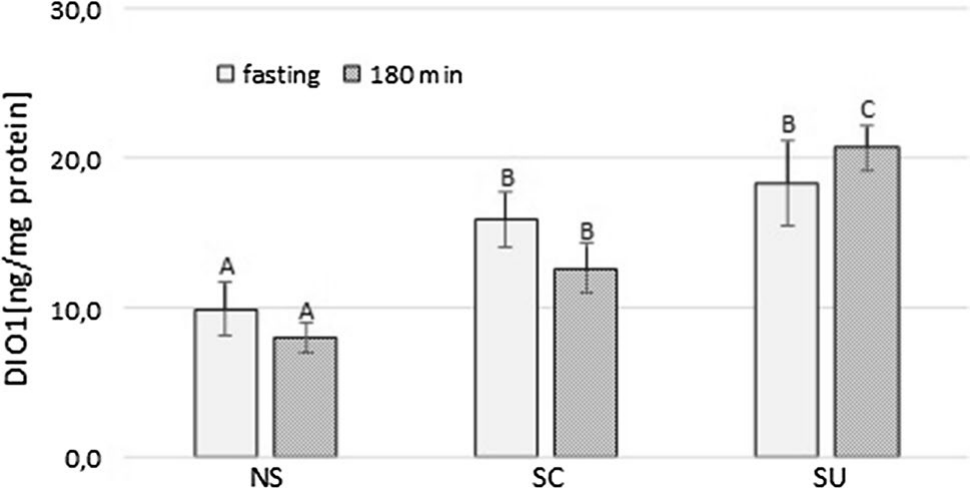
Deiodinase type 1 level in the liver (ng/mg protein) at fasting and 180 min after the meal; NS, non-sweet diet; SC, diet with sucrose; SU, diet with sucralose; *A*, *B*, *C* letters indicate significant differences between dietary groups (*p* < 0.05); data presented as mean ± SEM; *p < 0.05 compared to baseline

At fasting, the hepatic DIO1 level in NS was lower than in SC and SU (*p* = 0.011 and *p* < 0.001, respectively). Although the meal did not evoke any significant changes in the level of the hepatic enzyme, postprandial DIO1 in SU was significantly higher compared to NS (*p* < 0.001) and SC (*p* < 0.001) (Fig. 5).

Independently from the sampling period, a positive correlation between TSH concentration and T4 (*r* = 0.45, *p* = 0.035) was determined in SC only.

## Discussion

The hypothalamic-pituitary-thyroid axis response to alterations in the quantity and quality of the diet plays an essential role in the metabolic adaptation to ingested nutrients [25]. Carbohydrates have been shown to affect thyroid axis activity [13]; however, the effect of their non-nutritive substitutes on thyroid parameters remained as yet uninvestigated. In our study, three isoenergetic diets with equal levels of carbohydrates were applied: two with the addition of sweetness-matched sucrose and sucralose and the third one without a sweet flavour carrier.

The data on food intake, body weight gain, and diet efficiency presented in this study were previously discussed [26].

We have shown that both the presence of a sweet flavour and the type of its carrier affect thyroid axis activity at fast and postprandial state. As baseline TPO activity, TSH, T4, and T3 concentrations did not differ significantly in rats fed the non-sweet diet and in rats receiving the diet with sucrose, the effect of the sweet taste on those parameters may be excluded. Hence, lower levels of those indices in SU should be exclusively attributed to the presence of sucralose in the diet. Lower basal TSH followed by reduced TPO activity and plasma thyroid hormone levels might indicate a decreased central or peripheral feedback control and resembles the relations in HPT axis activity during starvation [27] or in the non-thyroidal illness syndrome [28]. It is noteworthy that lower levels of TSH and total thyroid hormones in the SU group occurred despite a higher daily food intake and weight gain than in the two other groups. This is inconsistent with the known positive relations between the amount of consumed food and the levels of circulating thyroid hormone [29] and might suggest that regular exposure to sucralose alters the thyroid axis response to ingested food.

Interestingly, lower levels of total T4 and T3 were accompanied by higher free-to-total thyroid hormone ratios in the group on the sucralose-added diet than in NS and SC. Although at fast, fT4 concentration was not influenced by the diet type, fT3 in SU remained at the highest level. Taking into consideration that free fatty acids (FFA) compete with T4 and T3 for binding sites on plasma transport proteins [30], a higher free-to-total hormone concentration ratio in SU might have resulted from a substantially higher postprandial lipaemia than in the two other groups.

On the other hand, the pattern of HPT axis components—decreased TPO activity, TSH, T4, and T3 plasma concentrations together with increased free-to-total TH ratios in the group on the diet with sucralose—resembles some effects evoked by organochlorine compounds documented in human and animal studies. The inverse relationships between plasma levels of chloroorganic compounds and TSH or the thyroid hormone have been observed [31–35]. The association between high levels of fT4 and the consumption of fish exposed to organochlorinated xenobiotics was found in adults from a certain area in East Slovakia [36]. This could be explained by the binding of chloroorganic compounds residues to transthyretin [37]. In the light of these parallels, our results could raise questions about the physiological inertness of sucralose.

Such doubts were already expressed by others on the basis of the influence of sucralose intake on the expression of rat intestinal P-glycoprotein (P-gp) and cytochrome P-450 isozymes, which are key components of the detoxification system in first-pass drug metabolism [38]. Alterations in beneficial intestinal microflora and epithelial border function after long-term sucralose ingestion were also recorded [38, 39]. However, in two-year carcinogenicity studies administration of sucralose in high doses was not connected with any sucralose-related neoplasms including thyroid tumours [40, 41]. Even though plasma thyroid profile was not included in those protocols, we could only assume that potential long-term deviations in thyroid axis activity would have been reflected in thyroid histopathology.

After the food was consumed, changes in the monitored parameters occurred only in the groups fed the sweetened diets and their postprandial time course of changes was affected by the type of sweet flavour carrier. Two contrary trends in TSH concentration might indicate distinct post-ingestion regulatory mechanisms. The meal-induced TSH decline observed in the sucrose-fed group was reported also by others [42–44]. Recently, the role of bile acids in the postprandial TSH decrease has been suggested [42]. The high expression of the bile acid receptor TGR-5 was found in the paraventricular and supraoptic nuclei of the hypothalamus in rats [45]. This might indicate that bile acid–TGR-5 signalling regulates thyroid axis activity by a direct modulation of TRH and TSH release. However, in our study the same fat content and fatty acid composition, lower daily food and, consequently, fat intake in SC compared to SU eliminate the impact of bile acids on the differentiation of TSH response to meal intake. Therefore, we are likely to presume that a more efficient negative feedback mechanism was involved in the post-ingestive TSH decline in SC as the postprandial T3 concentration in animals on the diet with sucrose was markedly higher than in SU.

Distinct neuronal [46–48] and hormonal [49, 50] reactions after sucrose and sucralose intake should also be taken into account. Although sweet taste perception of both these tastants is mediated by heterodimeric T1R2 and T1R3 gustducin-coupled receptors present in the oral cavity, sucralose binds to taste receptor subunits with greater affinity than sucrose [51]. This finding might have a profound metabolic implication since sweet taste receptors have also been identified in the brain, pancreas, and gastrointestinal tract [20, 21]. It is worth pointing out that taste genes are highly expressed in paraventricular nuclei of the hypothalamus—a brain region fundamentally implicated in thyroid hormone feedback regulation [52]. The higher expression level of the *Tas1r2* gene in cultured hypothalamus cells exposed to a hypoglycaemic medium and the lower transcript level observed after the exposition to a higher concentration of glucose indicate that the taste signalling mechanism in the brain is regulated by a nutritional status and could be involved in the central control of energy homoeostasis. Interestingly, the addition of sucralose into a low-glucose medium elicited a greater *Tas1r2* expression decline than hyperglycaemic conditions, indicating that the taste-like signalling in hypothalamic neurons does not require an intracellular increase in glucose metabolism [52]. Therefore, it was suggested that the stimulation of taste receptors in nutrient-sensing brain areas by artificial sweeteners might impair the central mechanism regulating glucose metabolism [52].

Furthermore, it has been established that sucrose and sucralose impact differently the reward system—dopaminergic midbrain areas that play an essential role in motivated behaviour. Frank et al. [47] demonstrated, using functional magnetic resonance imaging, that although both 10% sucrose and an equisweet sucralose solution activated neuronal taste pathways, sucrose more strongly induced the reward-related regions of the brain. Moreover, sucrose, but not sucralose, was able to induce dopamine release in mice lacking functional sweet taste transduction (*trmp5* ^−*/*−^), which suggests that the dopamine-related brain reward system responds to metabolic signals rather than gustatory inputs [46] and the glucose oxidation rate independently of sweetness perception plays a key role in dopamine release [48]. This indicates that the reward system acts as a metabolic sensor regulating sugar-specific behavioural preferences. Thus, the diet with added sucrose and probably a higher glucose oxidation rate than the diet with starch accompanied with non-metabolisable sucralose might influence thyroid axis activity through the modulation of brain dopamine circuits as it is known that dopamine administration suppresses TSH release [53].

Besides glucose-containing sugars that have a dominant role in dopamine release in a concentration-dependent manner [54], midbrain dopamine neurons express insulin [55], leptin [56], and glucagon-like peptide 1 (GLP-1) receptors [57]. The secretion of gut hormones including GLP-1, glucose-dependent insulinotropic peptide (GIP), cholecystokinin (CCK), and peptide YY (PYY) is induced by the sweet taste receptor heterodimer T1R2/T1R3 expressed in intestinal enteroendocrine cells in response to the presence of tastants in the lumen [58, 59]. Although in vitro studies conducted on the human L cell line NCI-H716 have shown that both sucrose and sucralose stimulate GIP and GLP-1 secretion [60], in clinical trials oral [49] and intragastric infusion [50, 61] of sucralose solutions did not affect insulin, GLP-1, and PYY secretion. In our study, the insulin level was found to be lower in the group fed the diet with sucralose than sucrose despite the fact that both were accompanied by starch [26]. Hence, those observations provide indirect evidence of a lower GLP-1 level in SU which is responsible for approximately 50–70% of postprandial plasma insulin increase [50]. Since GLP-1 [62] and peptide YY [63] were shown to modulate TSH secretion, various effects of sucrose and sucralose on incretins release could contribute to the observed differences in the TSH response to a meal.

Similarly, as in the case of TSH, meal ingestion evoked TPO activity changes only in animals fed the sweetened diets. In both SC and SU, increases in postprandial TPO activity were recorded; however, the peak times were different. This rapid growth of TPO activity in rats fed the sucralose-added diet might result from post-translational modifications combined with a movement to the follicular cell apical membrane and the formation of a multiprotein complex involved in thyroid hormone biosynthesis [64]. However, striking is the fact that TPO activity increase in SU was accompanied by the lowest T4 level during the entire sampling period.

Despite the known species derived differences in thyroid economy between humans and rodents [65, 66], it was demonstrated that total T4 levels in rodents are a valid indicator of thyroid function in relation to effects in humans [67]. Moreover, humans and rats might be equally sensitive to TH synthesis disruptors, and even though in rats the response occurs after a shorter exposure time, the final effect could be the same [68].

The changes in TPO activity in both groups in the monitored time points are difficult to link with the changes in the TSH plasma concentration. We presume the impact of other factors on thyroid activity including insulin, insulin-like growth factor-1 (IGF-1) [69], norepinephrine, acetylcholine, and peptides—neuropeptide Y (NPY), vasoactive intestinal peptide (VIP), substance P, CCK, or gastrin [70]. Interestingly, it was recently shown that bitter taste receptors are also expressed in thyrocytes. They negatively regulate TSH-dependent Ca^2+^ signalling, TSH-dependent iodide efflux, TPO activity, and TH production. Therefore, it is suggested that receptors activated by bitter-tasting stimuli might be involved in the protective response to an overdose of toxic substances [23]. Up to date, there have been no available data on the relationship between sweet taste receptors and the thyroid function.

The hepatic DIO1 level seemed to be increased by a sweet taste as liver enzyme content was lower and the rT3 plasma concentration was higher in rats fed the non-sweet diet. The lower T3/T4 ratio and the DIO1 level in the liver of rats fed the non-sweetened diet suggest a reduced 5′-monodeiodination efficiency. Since glucose and sucrose have been shown to stimulate DIO1 activity [13, 71], it is tempting to imply the role of extraoral sweet taste receptors in these effects.

Although the postprandial increase in the enzyme protein level was observed only in rats on the sucralose-added diet, the higher T3 baseline concentration and the post-meal T4 decrease in SC suggest greater hepatic 5′-deiodination efficiency which presumably was caused by higher enzyme activity. Consequently, these observations support the previously demonstrated stimulatory effect of sucrose on hepatic T4 to T3 conversion [13, 72].

In summary, the sweet taste and its flavour carrier have a significant impact on pituitary-thyroid axis activity both at fast and postprandially. We showed that the effects promoted by the non-sweet diet are different from those of the sweetened diet, wherein the diets with sucrose and sucralose elicit opposite responses. In NS, the lowest hepatic DIO1 level and the highest rT3 at fasting and after feeding indicated diminished metabolism of the peripheral thyroid hormone. Given the TPO activity, hepatic DIO1 level, and TH profile, it could be concluded that the addition of sucrose accelerates thyroid axis activity. In turn, a daily intake of sucralose with the diet at doses close to the recommended ADI might disrupt thyroid hormone synthesis as TPO activity, T4, and T3 plasma concentrations in rats demonstrated the lowest levels.

## Conclusion

One principal finding of this study concerns the close relationship between the sweet flavour carrier and the pituitary-thyroid axis activity, which is involved in the metabolic adaptation to meal composition. This effect may be observed at various levels. Sucralose intake seems to diminish thyroid axis activity by decreasing TPO activity, TSH, and plasma total TH concentrations, but at the same time, it increases both free T3 and T4 indexes. Those findings confirmed that sucralose is physiologically active and may provoke disturbances in thyroid axis activity. Aware of uncertainties in the interpretation and extrapolation of data from laboratory animals to humans, we acknowledge that further studies are required to support these findings.
